# Tryptophan metabolism, its relation to inflammation and stress markers and association with psychological and cognitive functioning: Tasmanian Chronic Kidney Disease pilot study

**DOI:** 10.1186/s12882-016-0387-3

**Published:** 2016-11-10

**Authors:** Naama Karu, Charlotte McKercher, David S. Nichols, Noel Davies, Robert A. Shellie, Emily F. Hilder, Matthew D. Jose

**Affiliations:** 1 ACROSS, School of Physical Sciences, University of Tasmania, Hobart, Tasmania Australia; 2 Menzies Institute for Medical Research, University of Tasmania, Hobart, Tasmania Australia; 3 Central Science Laboratory, University of Tasmania, Hobart, Tasmania Australia; 4 School of Medicine, University of Tasmania, Hobart, Tasmania Australia; 5 Renal unit, Royal Hobart Hospital, Hobart, Tasmania Australia; 6 Present address: The Metabolomics Innovation Centre (TMIC), Department of Biological Sciences, University of Alberta, Edmonton, Alberta T6G 2E9 Canada; 7 Present address: Trajan Scientific and Medical, 7 Argent Place, Ringwood, Victoria 3134 Australia; 8 Present address: Future Industries Institute, University of South Australia, Mawson Lakes Campus, GPO Box 2471, Adelaide, South Australia 5001 Australia

**Keywords:** Chronic kidney disease, Tryptophan, Kynurenine, Neopterin, Cortisol, Inflammation, Depression, Anxiety, Cognition

## Abstract

**Background:**

Adults with chronic kidney disease (CKD) exhibit alterations in tryptophan metabolism, mainly via the kynurenine pathway, due to higher enzymatic activity induced mainly by inflammation. Indoles produced by gut-microflora are another group of tryptophan metabolites related to inflammation and conditions accompanying CKD. Disruptions in tryptophan metabolism have been associated with various neurological and psychological disorders. A high proportion of CKD patients self-report symptoms of depression and/or anxiety and decline in cognitive functioning. This pilot study examines tryptophan metabolism in CKD and explores associations with psychological and cognitive functioning.

**Methods:**

Twenty-seven adults with CKD were part of 49 patients recruited to participate in a prospective pilot study, initially with an eGFR of 15–29 mL/min/1.73 m^2^. Only participants with viable blood samples and complete psychological/cognitive data at a 2-year follow-up were included in the reported cross-sectional study. Serum samples were analysed by Liquid Chromatography coupled to Mass Spectrometry, for tryptophan, ten of its metabolites, the inflammation marker neopterin and the hypothalamic–pituitary–adrenal (HPA) axis marker cortisol.

**Results:**

The tryptophan breakdown index (kynurenine / tryptophan) correlated with neopterin (Pearson *R* = 0.51 *P* = 0.006) but not with cortisol. Neopterin levels also correlated with indoxyl sulfate (*R* = 0.68, *P* < 0.0001) and 5 metabolites of tryptophan (*R* range 0.5–0.7, all *P* ≤ 0.01), which were all negatively related to eGFR (*P* < 0.05). Higher levels of kynurenic acid were associated with lower cognitive functioning (Spearman *R* = −0.39, *P* < 0.05), while indole-3 acetic acid (IAA) was correlated with anxiety and depression (*R* = 0.52 and *P* = 0.005, *R* = 0.39 and *P* < 0.05, respectively).

**Conclusions:**

The results of this preliminary study suggest the involvement of inflammation in tryptophan breakdown via the kynurenine pathway, yet without sparing tryptophan metabolism through the 5-HT (serotonin) pathway in CKD patients. The multiple moderate associations between indole-3 acetic acid and psychological measures were a novel finding. The presented pilot data necessitate further exploration of these associations within a large prospective cohort to assess the broader significance of these findings.

**Electronic supplementary material:**

The online version of this article (doi:10.1186/s12882-016-0387-3) contains supplementary material, which is available to authorized users.

## Background

Tryptophan metabolism as well as inflammation and oxidative stress have been associated with various conditions, including psychiatric disorders [1–6], neurological diseases [7–9], chronic kidney disease (CKD), the early onset of cardiovascular disease (CVD) [10–14], and even kidney allograft rejection [15]. The progression of CKD to end-stage renal disease (ESRD) is often accompanied by a decline in health-related quality of life involving an increase in symptoms of depression, anxiety and a deterioration in cognitive functioning [16–18]. In CKD patients these problems are less commonly identified, investigated and managed, partly due to the overlap between psychological symptoms and the symptoms of uraemia. Nevertheless, these symptoms affect the frequency of hospitalisation, adherence with treatment and play a role in the progression of the disease and mortality [16, 17, 19]. Early intervention is important, since inflammation and oxidative stress are already evident in the moderate stages of CKD [20], and these factors enhance the metabolism of tryptophan via the kynurenine pathway. In normal health, the majority of peripheral tryptophan is degraded in the liver via the kynurenine pathway by tryptophan 2,3-dioxygenase (TDO), controlled mainly by tryptophan levels. A further shift towards the kynurenine pathway occurs in the case of inflammation, such as in CKD [21] or the chronic low-grade inflammation in elderly people [6]. Pro-inflammatory molecules, especially the cytokine IFN-γ, activate the enzyme indoleamine-(2,3)-dioxygenase (IDO) in extra-hepatic tissues [22] and also promote the production of neopterin, a sensitive immune-response marker. When cytokines activate the hypothalamo-pituitary-adrenal (HPA) axis, the anti-inflammatory glucocorticoids produced also enhance the activity of TDO [1, 7]. Psychological disorders may be explained by disruptions in tryptophan metabolism in two directions [1]. The first is the high activity of IDO and TDO, which promote tryptophan breakdown via the kynurenine pathway, depriving tryptophan hydroxylase (TPH) of its substrate in the 5-HT (5-hydroxy tryptamine, serotonin) pathway, resulting in reduced serotonin production [5, 23–25]. The second direction is the neuroactivity of the kynurenine pathway metabolites (kynurenines) [2, 4, 26, 27], which can originate in peripheral kynurenine crossing the blood–brain barrier (BBB), thereby acting independently of brain tryptophan levels. Oxidative stress and inflammation not only promote production of kynurenines, but are also induced by some of the kynurenines (i.e. 3-OH Kynurenine and quinolinic acid) [1, 2]. Therefore, these metabolites can contribute non-specifically to different symptoms, with some kynurenines such as quinolinic acid and kynurenic acid also possessing specific neuroactivity [7, 9, 24, 28]. Another type of potentially toxic tryptophan metabolites are uraemic indoles produced by gut microflora [29–31]. These include indoxyl conjugated to sulfate, which is associated with ROS formation, CVD and progression of glomerular sclerosis [13, 32–34]. The symptoms of uraemia are affected by the different extent of metabolite accumulation and varying clearance rates. For example, uraemic accumulation (compared to normal blood) can be as high as 60-fold for neopterin, 43-fold for indoxyl sulfate, and 27-fold for kynurenic acid [35]. As part of a preliminary investigation of serum metabolomics we identified an array of tryptophan metabolites differentiating between haemodialysis patients and healthy volunteers, as well as between pre- and post-dialysis samples [36]. These findings, in agreement with literature values where available [11, 14, 35, 37] led to the selection of candidate metabolites for the current study. The study includes the sensitive measurement of serum metabolites by High-Performance Liquid Chromatography (HPLC) coupled to Mass Spectrometry (MS).

The present study aims to provide preliminary data regarding associations between kidney function and three elements: tryptophan metabolism, markers for inflammation and oxidative stress, and psychological/cognitive functioning. Previously published work has mostly linked pairs of these three elements, as discussed earlier. Our hypotheses were as follows: (1) neopterin and/or cortisol are related to tryptophan metabolism and (2) these markers and/or tryptophan metabolites relate to the severity of psychological symptoms. The long-term goal of this research is to improve psychological and cognitive functioning in patients with CKD by minimising neurotoxic responses. So far, studies have examined the effects of psychopharmacological treatments on psychological symptoms (see review [38]), adherence with haemodialysis treatment and clinical outcomes. In the complex case of CKD, additional factors should be taken into account, however there is still insufficient evidence supported by quantitative studies of relevant biomarkers and the affected metabolic pathways.

## Methods

### Participants

The protocol for the Tasmanian Chronic Kidney Disease study has been published previously [39]. Briefly, clinical data and serum samples were obtained from 27 patients who were recruited 2 years earlier (while with CKD stage 4) via the treating physician, to participate in a prospective cohort pilot study. The ensuing large-scale study aims to examine the influence of both biomedical and psychosocial factors on disease progression, decision making and length and quality of life in adults residing in Tasmania, Australia, with severe CKD and prior to kidney replacement therapy.

### Procedure

Participants were sent self-report questionnaires including socio-demographic information, psychological and cognitive functioning, prior to attending a study clinic where baseline clinical and laboratory data were collected. Participants unable to attend a clinic had measurements taken at their place of residence. Serum samples from dialysis patients were collected just prior to dialysis treatment. Samples were stored at −80 °C until analysis by liquid chromatography-tandem mass spectrometry (LC-MS/MS). Only participants with viable serum samples and complete psychological / cognitive data at follow-up (*n* = 27) were included in the current study.

### Biomedical factors

eGFR was calculated using the CKD-EPI formula from serum creatinine quantified by enzymatic assay and IDMS-aligned. Body mass index (BMI) (kg/m^2^) was calculated from objectively measured height and weight. Arterial hypertension was defined as systolic blood pressure ≥140 mmHg, diastolic blood pressure ≥ 90 mmHg, or patient prescribed antihypertensive medication.

### Psychological and cognitive functioning

Depression and anxiety were self-reported using the Patient Health Questionnaire (PHQ-9) and the Beck Anxiety Inventory (BAI). The PHQ-9 is a screening measure for clinical depression and includes nine items. Respondents self-report how often they experienced each item during the previous fortnight (between 0, never; to 3, nearly every day). The threshold for clinical depression in haemodialysis patients is a score of 10 and above [40], while levels of 5–9 are generally regarded as mild depression. The BAI was designed to distinguish between the symptoms of anxiety and those of depression. It consists of 21 items, each rated for the bothersomeness of the symptom in the last month, ranging from 0 (not at all) to 3 (severely). Out of the maximum score of 63, ≤ 7 is categorized as ’no anxiety‘, 8–15 ’mild anxiety‘, 16–25 ’moderate anxiety‘ and over 25 ’severe anxiety’ [41]. Participants also completed the cognitive function subscale of the Kidney Disease Quality of Life short-form (KDQOL-SF 1.3). The KDQOL-SF combines the 36 generic items of the SF-36 with 43 kidney disease-targeted items. Eleven subscales are defined from the kidney disease-targeted items, resulting in a total of 19 subscales (i.e., eight generic and 11 targeted to patients with kidney disease and treated by dialysis). Responses are weighted and transformed to scores ranging from 0 to 100, with higher scores indicating better self-assessed health-related quality of life.

### Chemicals

Reagents were purchased from Merck (Darmstadt, Germany) and chemical standards were from Sigma–Aldrich (WI, USA). A Milli-Q system (Millipore, MA, USA) was used to purify water for aqueous solutions.

### Quantification of serum metabolites

Serum was protein-precipitated with methanol (1:3 v/v), centrifuged at 16600x g for 15 min at 4 °C (Sigma 1-14 K Microcentrifuge, Germany), the collected supernatant dried, and reconstituted in water/acetonitrile (95:5 v/v) to the original concentration. Quality control (QC) sample was prepared from a pool of 30 serum samples. Quantitation was by calibration plots based on QC spiked with external calibration standards at a series of seven concentrations (8000-fold range) spanning physiological ranges (see Additional file 1: Table S1). Analytes were separated on a ZORBAX SB-C18 analytical column (3.5 μm, 3.0x150 mm; Agilent, CA, USA), utilising a Waters Acquity H-class UPLC system (Waters, MA, USA) coupled to a Waters Xevo triple quadrupole Mass Spectrometer (Waters, Manchester, UK). Chromatography and mass spectrometry conditions are detailed in an additional file (see Additional file 2). Peak integration was conducted using Waters MassLynx and TargetLynx software, followed by export to Microsoft Excel (Microsoft, WA, USA).

### Statistical analysis

Correlations between psychological and cognitive measures, metabolites and patient characteristics were estimated using Spearman rank analysis. Correlations between metabolites and also between metabolites and patient characteristics were measured by Pearson’s correlation test. Comparisons of mean psychological and cognitive scores between two groups were conducted using Mann–Whitney U-test. Quantified metabolites were compared by Student’s *t-*tests or Welch’s ANOVA with Games-Howell post-hoc test. Correction for multiple hypotheses was applied using Benjamini- Hochberg false discovery rate (FDR). Prior to parametric tests on quantified metabolites, data were log-transformed and normal distribution confirmed by Shapiro-Wilk test. A two-tailed *P* value < 0.05 was considered statistically significant. Statistical analysis was conducted using XLStat V. 2014.3.04 (Addinsoft, Paris, France) and network analysis utilized the software Vanted V.2.1.0 (IPK, Gatersleben, Germany) [42].

## Results

### Demographic and clinical data of CKD patients

Clinical and demographic characteristics are detailed in Table 1. Kidney function at the 2-year follow up examined in this study, ranged from kidney failure (52 % at stage 5 CKD: eGFR < 15 mL/min/1.73 m^2^ or on haemodialysis) to severe CKD (37 % at stage 4: eGFR 15–29 mL/min/1.73 m^2^) and a few moderate CKD (11 % at stage 3b: eGFR > 29 mL/min/1.73 m^2^).

**Table 1:** Demographic and clinical characteristics of Tasmanian CKD patients participating in the study (*n* = 27)

Parameter	Frequency (%) or Mean ± SD [Range]
Male (%)	18 (67 %)
Age (years)	76.4 ± 7.3 [60–87]
Education > Year 12	9 (33 %)
Living with a partner	22 (81 %)
Retired	23 (85 %)
Smoker, current or former	16 (59 %)
Body mass index (kg/m^2^)	29.8 ± 5.1 [21.5–42.4]
Serum creatinine (μmol/L)	336.4 ± 178.2 [121–863]
eGFR (mL/min/1.73 m^2^)	17.0 ± 8.4 [5–36]
CKD	
Stage 3b	3 (11 %)
Stage 4	10 (37 %)
Stage 5 (non-dialysed)	7 (26 %)
Stage 5 (haemodialysis)	7 (26 %)
Depression score (PHQ-9)	3.5 ± 3.5 [0–14]
Anxiety score (BAI)	7.2 ± 6.4 [0–25]
Cognitive functioning (in KDQOL-SF 1.3)	86.2 ± 15.5 [46.7–100]
≥3 comorbidities	14 (52 %)
Diabetes mellitus	7 (26 %)
Hypertension	21 (78 %)
Atherosclerotic heart disease	9 (33 %)
Congestive heart failure	9 (33 %)
Peripheral vascular disease	7 (26 %)
Prescribed antidepressants	3 (11 %)
Prescribed ACE inhibitors	8 (30 %)
Prescribed Angiotensin II Receptor Blockers	8 (30 %)
Prescribed statins	18 (67 %)
Prescribed antibiotics	2 (7 %)

*Abbreviations*: *ACE* angiotensin-converting enzyme, *BAI*, Beck Anxiety Inventory, *CKD* chronic kidney disease, *eGFR* estimated glomerular filtration rate, *KDQOL-SF*, Kidney Disease Quality of Life short-form, *PHQ-9*, Patient Health Questionnaire

### Tryptophan metabolism and association with clinical factors

**Fig. 1:**
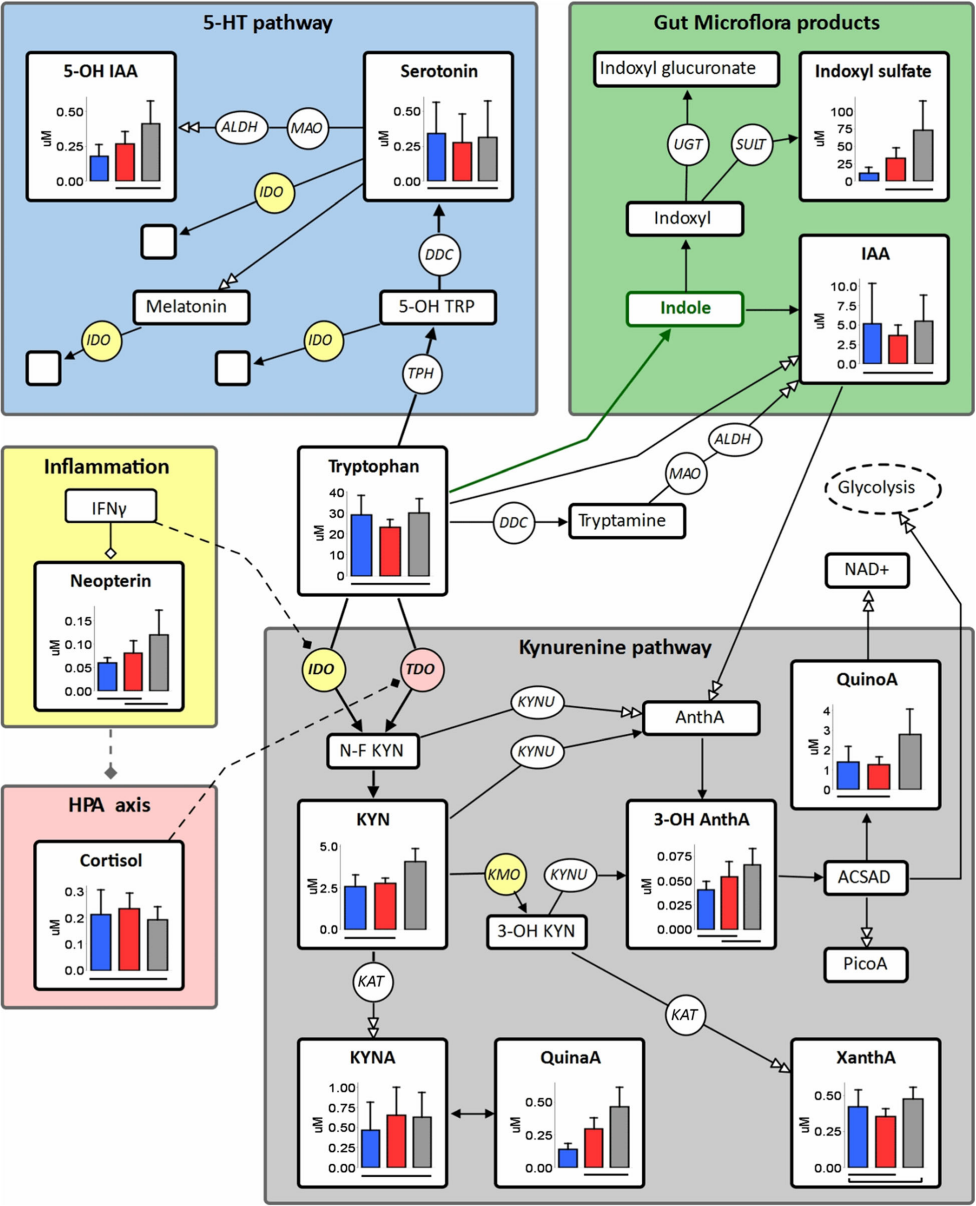
A schematic diagram of Tryptophan metabolism, including box plots of quantified metabolites. Metabolites are in *squares*, enzymes in *circles* (*yellow* affected by cytokines, *pink* by cortisol), *double-tip arrow* indicates multi-step metabolism, *Green arrow* and metabolite are produced only by gut microflora. Box plots depict mean levels ± s.d (μM) grouped by kidney function, from *left* to *right*: *blue*, 15 ≤ eGFR ≤ 36 (CKD stage 3b-4, *n* = 13); *red*, eGFR < 15 (CKD stage 5, non-dialysis); *grey*, Haemodialysis patients (CKD stage 5). For each metabolite, a *horizontal line* under a pair of box plots indicates a non-significant difference in mean values (Welsh’s one-way ANOVA with Games-Howell post-hoc test, *P* > 0.05). Abbreviations: ACSAD, 2-amino-3-carboxymuconate semialdehyde; ALDH, aldehyde dehydrogenase; Anth A, anthranilic acid; CKD, chronic kidney disease; DDC, aromatic amino acid decarboxylase; eGFR, estimated glomerular filtration rate; IAA, indole-3-acetic acid; IDO, indoleamine-(2,3)-dioxygenase; IFNγ, interferon-γ; KAT, kynurenine aminotransferase; KMO, kynurenine 3-monooxygenase; KYN, kynurenine; KYNA, kynurenic acid; KYNU, Kynureninase ; MAO, monoamine oxidase ; NAD+, Nicotinamide adenine dinucleotide; N-F, N-formyl; PicoA, picolinic acid; QuinaA, quinaldic acid; QuinoA, quinolinic acid; SULT, sulfotransferase; TDO, tryptophan 2,3-dioxygenase; TPH, tryptophan hydroxylase; UGT, UDP glucuronosyltransferase; XanthA, xanthurenic acid

**Fig. 2:**
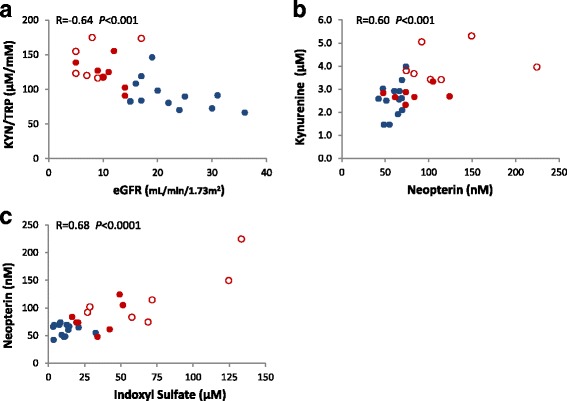
Scatter plots depicting relationships between serum metabolites. **a** tryptophan breakdown index (KYN/TRP) vs. eGFR; **b** kynurenine vs. neopterin; **c** neopterin vs. indoxyl sulfate. Marker colours indicate kidney function, and not used for separate calculations: red, CKD stage 5 (empty, HD; full, non-HD); blue, CKD stage ≤ 4. Pearson correlation tests (*n* = 27) were conducted on log-transformed data as required, then back-transformed for presentation in this figure. Abbreviations: CKD, chronic kidney disease; eGFR, estimated glomerular filtration rate; HD, haemodialysis; KYN, kynurenine; TRP, tryptophan

Table 2 summarizes the LC-MS/MS quantified levels of tryptophan, ten of its metabolites, and also the inflammation marker neopterin and the HPA-axis activity marker cortisol. Uraemic and normal reference levels are also presented in the table, to support the credibility of the quantified levels. The variability in uraemic symptoms observed in the patients is not unexpected, as serum levels depend on renal function (or dialysis treatment), as well as on gender, genetic factors, diet and also gut microflora [37]. This is reflected in the high levels and ranges of the gut-microflora produced metabolites examined here (indoxyl sulfate and indole-3 acetic acid, IAA). No correlations were found between the patients’ BMI and any of the examined metabolites, as shown in Table 3A which summarizes Pearson correlations between metabolites and clinical factors. While eGFR and serum creatinine levels were not related to age, low correlation was observed between age and tryptophan levels (*R* = 0.46, *P <* 0.05). A negative relation was also found between age and the tryptophan breakdown index KYN/TRP, which is the rate of tryptophan conversion to the uraemic molecule kynurenine (*R* = −0.44, *P <* 0.05). Although age did not show any correlation with serotonin, it was inversely related to the serotonin turnover metabolite 5-hydroxy-3-indole acetic acid (5-OH IAA, *R* = −0.55, *P* < 0.01). The quantified metabolites were put into biochemical context via the schematic overview of tryptophan metabolism illustrated in Fig. 1. The flow chart also contains box-plots depicting the mean serum metabolite levels (in μM) in patients grouped according to kidney function, to better represent similarities and trends. Patients receiving haemodialysis did not have reduced levels of tryptophan metabolites pre-dialysis compared to non-dialysed patients with eGFR < 15. The haemodialysis patients showed higher levels of the uremic molecules quinolinic acid and kynurenine (Welch’s one-way ANOVA with Games-Howell post-hoc test, *P* < 0.01, significant after FDR correction for multiple hypotheses). In comparison, patients with eGFR ≥ 15 (CKD stages 4 and 3B) had lower levels of some of the metabolites. The relation to eGFR and creatinine is further described for the full cohort by Pearson correlation tests in Table 3. In agreement with previous studies, the tryptophan breakdown index, KYN/TRP, generally increased with deterioration in kidney glomerular function (*R* = −0.64, *P* < 0.001; Fig. 2a), and there was a moderate correlation between tryptophan and kynurenine (*R* = 0.56, *P* < 0.01). The two markers hypothesized to be related to this conversion via TDO and IDO enzymes were also examined. Cortisol, the HPA axis activity marker, was within the wide normal range in all patients and did not show significant associations with any of the quantified metabolites. On the other hand, the inflammation marker neopterin significantly increased as eGFR dropped (*R* = −0.56, *P* < 0.01). Although neopterin was not directly associated with tryptophan levels, it was significantly correlated with KYN/TRP (*R* = 0.55, *P* < 0.01), and showed moderate positive correlations with six tryptophan metabolites (see Table 3). These tryptophan metabolites include kynurenine (*R* = 0.60, *P* < 0.001*;* Fig. 2b) and three of its downstream metabolites, as well as indoxyl sulfate (*R* = 0.68, *P* < 0.0001*;* Fig. 2c). Moreover, moderate positive correlations were persistent between indoxyl sulfate and the kynurenines above, as well as KYN/TRP (*R* = 0.69, *P* < 0.0001). The second bacteria-produced tryptophan metabolite measured here, IAA, is a known uraemic molecule yet it did not correspond with eGFR or any of the metabolites, and was highly variable within the groups. Tryptophan can also be metabolized via the 5-HT (serotonin) pathway, as depicted in Fig. 1. According to our results, serum serotonin levels did not differ between kidney function groups or correlate with eGFR or any of the metabolites. The serotonin breakdown product 5-OH IAA, which is not considered a uraemic molecule, was negatively associated with eGFR (*R* = −0.67, *P* < 0.001). 5-OH IAA also highly correlated with KYN/TRP, and was moderately correlated with neopterin and indoxyl sulfate (see Table 3).

**Table 2:** Summary of serum metabolites quantified by LC-MS/MS, with reference values for healthy and uraemic blood

		Current study (*n* = 27)	Reference values^	
Metabolite	Units	Mean ± s.d. [range]	Healthy adult	Uraemic (CKD)
Neopterin^a^	nM	81.2 ± 38.2 [42.4–224.6]	5.5 ± 2.0^c^ 8.5 ± 4.4^d^	329.1 ± 42.7 (max)^c^ 172 ± 88.0^e^
Cortisol	nM	212.9 ± 75.8 [91.4–468.7]	320 ± 190^f^	n/a
Tryptophan	μM	27.8 ± 7.9 [10.0–48.5]	54.5 ± 9.7^f^ 31.2 ± 7.4^g^ 62.8 ± 9.3^d^	17.3 ± 9.7^g^ 28.6 ± 8.8^e^
Serotonin (5-HT)	nM	317.6 ± 221.2 [33.3–825.2]	740 ± 280^f,h^	n/a
5-OH IAA	nM	262.6 ± 143.1 [114.2–704.6]	51.6 ± 6.8^f^	n/a
Kynurenine^b^	μM	3.02 ± 0.91 [1.46–5.32]	1.6 ± 0.1^f^ 1.6 ± 0.9^g^ 2.5 ± 0.7^d^	3.3 ± 0.9^i^ 2.7 ± 1.4^g^ 4.46 ± 2.5^e^
Kynurenic acid^b^	nM	557.3 ± 336.8 [168.4–1349.0]	30 ± 7^c,f^ 28.6 ± 17.1^g^	800 ± 400 (max)^c,f^ 336.1 ± 72.6^g^
Quinolinic acid^b^	μM	1.72 ± 1.07 [0.52–4.97]	0.6 ± 0.3^i^ 0.26 ± 0.1^g^	9.0 ± 5.4^i^ 7.9 ± 2.8^g^
Xanthurenic acid	nM	417.5 ± 102.8 [207.9–637.2]	21.9 ± 0.98^f^	170.1 ± 60.0^f^
Quinaldic acid	nM	264.1 ± 164.1 [90.4–671.0]	n/a	n/a
3-OH Anthranilic acid	nM	50.8 ± 16.7 [26.5–95.8]	79 [15.0–209.0]^f^	n/a
Indoxyl sulfate^b^	μM	33.2 ± 33.9 [3.24–133.3]	2.5 ± 1.4^c^	108.4 ± 61.0^c^
IAA^b^	μM	4.9 ± 4.0 [1.3–20.9]	2.9 ± 1.7^c^	11.6 ± 2.2^c^

*Abbreviations*: *CKD* chronic kidney disease, *max* highest uraemic concentration recorded (presented in case mean uraemic concentration is not available), *n/a* data not available, *s.d.* standard deviation

^Values were converted to molarity where given in g/L

^a^water-soluble uraemic molecule; ^b^protein-bound uraemic molecule; ^c^Taken from [35], indicating total values (in case of protein-bound metabolites); ^d^taken from [6] for elderly people; ^e^haemodialysis patients [11]; ^f^HMDB website [71]; ^g^taken from [14] for plasma from healthy subjects or pre-haemodialysis patients; ^h^data for males, for females values are 970 ± 280 nM; ^i^ taken from [37]

**Table 3:** Pearson correlation matrix of metabolites and clinical data (*n* = 27)

	Neopterin	Cortisol	Tryptophan	Serotonin (5-HT)	5-OH IAA	5-OH IAA/5-HT	Kynurenine	KYN/TRP	Kynurenic acid	Quinolinic acid	Xanthurenic acid	Quinaldic acid	3-OH anthranilic acid	Indoxyl sulfate	IAA
A) Clinical															
Age	−0.17	−0.34	0.46^*^	−0.03	−0.55^**^	−0.26	−0.37	−0.44^*^	0.02	−0.15	0.19	−0.23	−0.22	−0.24	0.17
BMI	−0.23	0.32	−0.27	−0.10	−0.05	0.06	−0.25	0.03	0.06	0.14	−0.21	−0.26	−0.19	−0.18	−0.30
Serum creatinine	0.65^***^	−0.15	−0.10	0.04	0.66^***^	0.31	0.53^**^	0.71^***^	0.17	0.43^*^	0.12	0.71^***^	0.63^***^	0.78^***^	0.04
eGFR	−0.56^**^	0.07	0.16	0.001	−0.67^***^	−0.35	−0.46^*^	−0.64^***^	−0.11	−0.41^*^	−0.01	−0.63^***^	−0.51^**^	−0.71^***^	0.12
B) Metabolites															
Neopterin	1														
Cortisol	−0.13	1													
Tryptophan	0.12	−0.29	1												
Serotonin (5-HT)	0.11	−0.06	−0.05	1											
5-OH IAA	0.50^**^	0.05	−0.37	−0.02	1										
5-OH IAA/5-HT	0.17	0.08	−0.15	−0.86^***^	0.53^**^	1									
Kynurenine	0.60^***^	−0.28	0.56^**^	−0.03	0.39*	0.21	1								
KYN/TRP	0.55^**^	−0.04	−0.45^*^	0.03	0.83^***^	0.40^*^	0.46^*^	1							
Kynurenic acid	0.26	0.06	0.11	−0.16	0.07	0.18	0.32	0.17	1						
Quinolinic acid	0.52^**^	−0.01	0.05	−0.04	0.33	0.20	0.49^**^	0.44^*^	0.05	1					
Xanthurenic acid	0.21	−0.09	0.56^**^	0.005	−0.18	−0.10	0.37	−0.20	−0.05	0.39^*^	1				
Quinaldic acid	0.67^***^	0.05	0.05	0.10	0.64^***^	0.25	0.60^***^	0.61^***^	0.37	0.23	0.10	1			
3-OH Anth acid	0.71^***^	0.003	0.13	0.11	0.35	0.09	0.57^**^	0.46^*^	0.48^*^	0.49^**^	0.30	0.75^***^	1		
Indoxyl sulfate	0.68^***^	−0.15	−0.23	0.09	0.58^**^	0.22	0.44^*^	0.69^***^	0.22	0.50^**^	−0.06	0.71^***^	0.63^***^	1	
IAA	0.07	0.10	0.17	−0.20	0.01	0.17	0.24	0.07	0.14	0.05	0.22	0.09	0.20	−0.07	1

*Abbreviations*: *5-HT* serotonin, *Anth* anthranilic, *BMI* body mass index, *eGFR* estimated glomerular filtration rate, *IAA* indole-3-acetic acid, *KYN* kynurenine, *TRP* tryptophan

Significance levels: *P < 0.05; **P < 0.01; ***P < 0.001

### Association between serum metabolites and psychological/cognitive functioning

Cognitive functioning was assessed using the cognitive subscale of the KDQOL-SF 1.3, and the results are summarized in Additional file 3: Table S2A. The cognitive function subscale has been previously validated in patients with CKD with a median score of 87 [43]. Here, the overall median was 93 (*n* = 27), showing an increase from the median of 73 in haemodialysis patients (*n* = 7, mean = 75), through a median of 93 in non-dialysed patients with CKD stage 5 (*n* = 7, mean = 89) to a median of 100 in the patients with CKD stages 4 and 3b (*n* = 13, mean = 91). Of the serum metabolites quantified in this study, high levels of kynurenic acid were associated with low cognitive functioning (*R* = −0.39, *P* < 0.05).

Depressive symptoms at a mild-moderate level were endorsed by 9 patients (33 %), while 2 patients met the threshold for clinical depression. Additional file 3: Table S2B reports the proportion of patients endorsing each depressive symptom (yes/no), with fatigue (66 %), sleep problem (44 %) and poor appetite (26 %) being the most prevalent. Of note, anhedonia (loss of interest in previously rewarding or enjoyable activities) which is one of the core symptoms of clinical depression was endorsed by 26 % of patients. The clinical parameters were not associated with any depressive symptoms. Of the quantified metabolites, IAA was the only metabolite to show significant correlation with the PHQ9 overall score (*R* = 0.39, *P* < 0.05), owing to low-moderate associations with the subscales sleep (*R* = 0.49, *P* < 0.01) and depression mood (*R* = 0.50, *P* < 0.01) which also correlated with 3-OH anthranilic acid (*R* = 0.40, *P* < 0.05). The association between the serotonin turnover (5-OH IAA/5-HT) and the symptom of poor appetite was the strongest recorded of any PHQ9 subscale (*R* = 0.51, *P* < 0.01). Interestingly, within the group of patients with ESRD (CKD stage 5, *n* = 14), serotonin and 5-OH IAA/5-HT showed moderate association with the total PHQ9 (*R* = −0.55, *R* = 0.59, respectively. both *P <* 0.05), and with the anhedonia subscale (*R* = −0.59, *R* = 0.69, respectively. both *P <* 0.05).

Additional file 3: Table S2C displays the prevalence of anxiety as measured by the 21 subscales of the BAI test. Twenty patients (74 %) reported at least three symptoms of anxiety, with two patients being classified as experiencing “moderate anxiety” and seven patients experiencing “mild anxiety”. Similarly to depressive symptoms, IAA displayed the strongest associations with anxiety symptoms (5 subscales) and the only moderate significant correlation with the total score (*R* = 0.52; *P* < 0.01). Six additional quantified metabolites showed significant yet low-to-moderate associations with anxiety subscales which were reported by at least 30 % of patients. eGFR showed low negative association with the subscale of indigestion, while three metabolites were moderately correlated with this subscale. Again, within the group of patients with ESRD (*n* = 14) multiple associations were found between subscales of anxiety, and serotonin and its turnover. The correlations between 5-OH IAA/5-HT and eight anxiety subscales led to a stronger positive correlation with total BAI score (*R* = 0.70, *P* = 0.005). Of note, seven of these subscales were complementary to those correlated with IAA.

### Differences based on prescribed medication

Some pharmaceuticals, especially antibiotics, ACE inhibitors and statins, affect inflammation and oxidative stress. Therefore, the corresponding levels of metabolites and scores were also compared between patients based on prescribed medication. The group of eight patients prescribed ACE inhibitors showed marginally lower levels of quinolinic acid and 3-OH anthranilic acid (all *t*-test *P* < 0.05). This group had a slightly higher depression score (total PHQ9 mean of 5.6 vs. 2.6; Mann–Whitney *P* = 0.013) and a higher anxiety score (total BAI mean of 11.2 vs. 5.4; *P = 0.018*). For ARB (Angiotensin II Receptor Blockers) and statins, no significant associations with metabolite levels were observed. The group that was prescribed statins (*n* = 18) displayed significantly lower anxiety (total BAI score mean of 4.5 vs. 12.4; *P* = 0.003) and also better cognitive functioning (mean of 91 vs. 76; *P =* 0.02). Only two patients were prescribed antibiotics, hence no statistical investigation was followed. Although less reliable to interpret due to the limitations of size and multiple unbalanced factors, medication should be taken into account when planning a larger cohort study.

## Discussion

This pilot study brings together data from different research areas, exploring associations between CKD progression, tryptophan metabolites, markers for inflammation and HPA activity, and psychological/cognitive functioning. There are a number of limitations of the current analysis that should be acknowledged. As this study was originally designed as a pilot to assess the feasibility of a larger cohort study, a formal power calculation was not performed. Another substantial limitation of the study design is the lack of healthy controls, dictated by the design of the Tasmanian CKD pilot study, as described in published work [39]. Results should therefore be treated as preliminary and interpreted with caution. In addition, due to the cross-sectional analysis, the direction of causality cannot be inferred. Further, it should be noted that depressive and anxiety symptoms, and cognitive functioning were assessed via self-report rather than standardized clinical interviews. While this approach may underestimate the prevalence of these disorders, all the self-report measures used in the current study have been validated in renal populations and are brief and easy to administer and interpret.

Our findings of increased tryptophan catabolites with deteriorating kidney function were mostly expected according to the literature, where available (Table 2). As we hypothesized, the high levels of the inflammation marker neopterin correlated with the tryptophan to kynurenine breakdown index. Similar correlations between KYN/TRP and neopterin were previously shown in haemodialysis patients [11] and in cases of kidney allograft rejection [15]. This was attributed to IDO-induced activation through inflammation [1, 2, 5, 7], and is in agreement with a CKD study by Schefold et al. who correlated other inflammatory markers with KYN/TRP [21]. Neopterin correlations found in the current study also included metabolites downstream in the kynurenine pathway: Quinolinic acid (as seen in [21]), 3-OH anthranilic acid, and also quinaldic acid, the redox product of kynurenic acid (showed correlation in [21]). Cortisol exhibited only non-significant trend towards negative associations with tryptophan and kynurenine, thus in this cohort we do not support the hypothesis concerning its involvement in tryptophan metabolism to kynurenine via TDO activation. This observation may, however, reflect inconsistent cortisol levels in the small cohort due to medications (i.e. ACE inhibitors), circadian variations and possible glucocorticoids resistance in some patients [44]. IDO activity may be indirectly induced by indoxyl sulfate [31], which promotes inflammation and oxidative stress [33]. The positive correlation we found between neopterin and indoxyl sulfate (*R* = 0.68; *P* < 0.0001) along with correlations between indoxyl sulfate and four kynurenines may further strengthen its link to inflammation and kynurenines production. This aspect also warrants further investigation in larger studies. With regard to the negative association we reported between indoxyl sulfate and eGFR (*R* = −0.71; *P* < 0.0001), it is debatable whether to expect a relationship with eGFR, since its clearance may be ruled by tubular secretion [45–47] or by glomerular filtration [48]. Regardless of the clearance mechanism, its inverse relationship to eGFR was demonstrated in several studies and was related to acceleration in the progression of CKD [34]. The increased tryptophan degradation via the kynurenine pathway, as observed in CKD, was proposed to be responsible for disruptions in the 5-HT pathway due to the loss of the precursor tryptophan [5, 23–25]. Criticisms of this hypothesis include the observation that in depressed patients, despite the inflammatory phenotype, the depletion of tryptophan was without changes in kynurenine pathway metabolites, expression of IDO or even the serotonin transporter (SERT) [49]. In our preliminary study with the low number of mostly mild depressive symptoms expressed by participants, the induced tryptophan metabolism via the kynurenine pathway did not seem to hinder metabolism via the 5-HT pathway. It is unclear whether the elevated levels of 5-OH IAA can be attributed to higher serotonin turnover or to uremic accumulation of 5-OH IAA. Nevertheless, a 9-fold increase of 5-OH IAA in CKD patients compared to healthy controls has been recorded before [50], and with our observation of association with eGFR, it may support the use of 5-OH IAA as a uraemic marker in CKD.

The second hypothesis in our work suggested association between metabolites (mainly kynurenines) and psychological/cognitive measures, possibly due to their neuroactivity. Our results revealed only a few associations between metabolites and psychological scores and subscales. As suggested in a recent review by Lopresti et al. [51], this lack of associations can result from differences between studies (type and level of psychological symptoms; comorbidities; treatment; study design; sample size). It can also originate in the involvement of additional biochemical mechanisms, not examined here, which are of high impact in CKD patients. The frequency of cognitive impairment estimated in the current study followed the expected pattern matching kidney function and treatment [52]. A low yet significant negative association was found between kynurenic acid and cognitive function, similarly to other studies [7, 24, 53], which regarded kynurenic acid as neurotoxic rather than neuroprotective (as previously hypothesized [28]). Also, we found no associations with the ratio kynurenic acid/kynurenine, termed the “neuroprotective ratio” in depression studies [28]. The neurotoxicity of kynurenic acid was partly explained by the effects of kynurenines on the nicotinic cholinergic system [54]. Kynurenic acid is a NMDA (N-Methyl-D-aspartate) receptor antagonist [5, 9, 28], unlike quinolinic acid, a NMDA receptor agonist which causes excitotoxic neurodegenerative changes [4, 7]. However, kynurenic acid has higher affinity to α-7-nicotinic acetylcholine receptors [4, 24, 54] and this can change its activity from neuroprotective to neurotoxic. The haemodialysis clearance of kynurenic acid, along with other kynurenines, is still insufficient despite advances in dialysis [12, 14, 21, 36]. The development of cognition-enhancement medication is an ongoing effort to address brain diseases, and these include pharmaceuticals that reduce the formation of kynurenic acid in the brain. Different approaches have been taken, for example by specific targeting of kynurenine aminotransferase II (KAT II, see Fig. 1) [55], which showed improved cognitive behaviour in mice lacking the expression of the enzyme [56]. Currently there is no standard treatment to eliminate kynurenines, and this emphasizes the need to reduce kynurenines formation via control of inflammation and other factors which contribute to tryptophan catabolism into kynurenines, including reduced consumption of tryptophan. An example for one approach is the treatment with selective vitamin D receptor activators (such as paricalcitol), which reduced inflammation and oxidative stress in renal patients undergoing haemodialysis [57]. Contrary to our hypothesis, mainly non-kynurenines were associated with depression and anxiety scales in our study. The self-reported complaints were mainly of low or mild symptoms, thus differing from other studies investigating tryptophan metabolism, especially in the field of psychiatry and brain diseases. Tryptophan itself did not correlate with any of the subscales, agreeing with a similar observation for a single subscale reflecting fatigue (SF-36 vitality) in haemodialysis patients [58]. 5-OH IAA along with serotonin turnover (5-OH IAA / 5-HT), were suggested before to serve as markers for evaluation of depression [59, 60]. The two markers were associated with a few subscales of depression and anxiety in our study. The multiple associations with subscales and also with total anxiety and depression scores found in patients with ESRD are of low statistical power. Nevertheless, the 5-HT pathway should not be neglected in future CKD studies in this context, also following evidence that serotonin is decreased in haemodialysis [21, 36], and data supporting the role of melatonin in fatigue symptoms of people with CKD [25]. Current guidelines recommend the use of an SSRI (selective serotonin reuptake inhibitor) as a first-line agent where treatment with antidepressants is considered in CKD patients [38]. Still, there is a lack of well-controlled trials that support or refute the efficacy and safety of antidepressant medications in patients with CKD [38, 61, 62]. This gap in medical evidence contributes to the status of clinical depression as under-recognized and an under-treated problem in this patient population. The tryptophan metabolites involving gut microflora gained our attention due to some surprising findings. Despite very high serum levels and extensive inter-metabolite correlations, indoxyl sulfate was only associated with one subscale of anxiety and not with any depressive symptoms. This, unfortunately, prevents a comparison with the intriguing report of lower serum indoxyl sulfate in depressed haemodialysis patients (compared to non-depressed patients) [63]. A novel finding in this pilot study is the role of indole-3-acetic acid (IAA) as the major metabolite related to both anxiety and depressive symptoms in CKD patients. IAA is a known uraemic molecule, and although its levels were not associated with serum creatinine or eGFR, they were elevated in some patients. IAA can be generated in the intestines from indole produced by gut microflora [30], or metabolized in tissue from tryptamine [64] and other tryptophan derivatives. IAA at uremic concentrations was previously linked to oxidative stress and contribution to development of CVD in CKD patients via activation of the Aryl Hydrocarbon Receptor [13]. The only literature we found relating IAA to psychiatry research included early studies in severely depressed patients, showing no change in urinary excretion of IAA throughout the stages of disease and recovery, despite a drop in tryptamine excretion [65] or plasma tryptophan [64]. In our work, the minimal relation between IAA and all other measured metabolites suggests the involvement of additional metabolic transformations not examined here, or subject-specific differences in its production and catabolism which obscured significant findings. Haemodialysis is rather limited in improving the clearance of indole-based uraemic toxins produced by gut microflora [33], depending on their conjugates and affinity to albumin. The intestinal absorption of indoles may be reduced by the orally-administered carbon-adsorbent AST-120. Adding this pharmaceutical to the standard therapy of CKD patients showed reduced serum levels of indoxyl sulfate [32, 66], improvement in some of the uraemia symptoms [66] and a decrease in markers for cardiovascular disease [67]. Although clinical trials showed that treatment with AST-120 resulted in more gradual disease progression [66, 68], its overall benefit is yet to be proven [68, 69]. Other approaches to reducing gut-microflora-produced uraemic molecules are consumption of a low-protein diet and restoring the balance of gut microflora by probiotics and prebiotics [30, 34, 70]. It would be interesting to examine the effects of such approaches on patients in various stages of CKD (including transplant and haemodialysis patients), and measure uraemic indole derivatives, tryptophan metabolites, inflammation markers and psychological variables.

## Conclusions

Our observations suggest that a decline in kidney function is associated with an increase in the inflammation marker neopterin and metabolism of tryptophan via the kynurenine pathway, without evident elimination of tryptophan metabolism via the 5-HT pathway. We also found along the continuum of CKD significant associations between neopterin, indoxyl sulfate, kynurenine and its downstream metabolites, as well as several associations with cognitive function and subscales of depression and anxiety. Of the quantified metabolites, the non-kynurenine IAA displayed consistent associations with symptoms of anxiety and depression, reported mostly at low to mild levels in the study. These pilot data suggest that further more detailed investigation of these associations is warranted. Exploration within a larger prospective cohort may provide a novel direction in improving psychological and cognitive well-being in this patient population.

## Additional files

Additional file 1:
**Table S1.** Concentration range of spiked analytes in calibration mix. (XLS 20 kb)
Additional file 2: Chromatography and mass spectrometry conditions. (PDF 276 kb)
Additional file 3:
**Table S2.** Spearman rank correlation coefficients for associations of psychological and cognitive functioning, with clinical parameters and serum metabolites. (XLS 36 kb)
Additional file 4: Raw Data in five data sheets including Patients relevant metadata, quantified metabolites (given at μg/L as well as μmol/L), psychology measures, common medication and comorbidities. (XLS 58 kb)

## Abbreviations

5-HT: 5-hydroxy tryptamine (serotonin)
5-OH IAA: 5-hydroxy-3-indole acetic acid
ACE: Angiotensin-converting-enzyme
ARB: Angiotensin II Receptor Blockers
BAI: Beck Anxiety Inventory
BBB: Blood–brain barrier
BMI: Body mass index
CKD: Chronic kidney disease
CVD: Cardio vascular disease
eGFR: Estimated glomerular filtration rate
ESRD: End-stage renal disease
FDR: False discovery rate
HPA: Hypothalamo-pituitary-adrenal
IAA: Indole-3-acetic acid
IDO: Indoleamine-(2,3)-dioxygenase
KDQOL-SF: Kidney Disease Quality of Life short-form
KYN/TRP: Kynurenine/tryptophan
KYN: Kynurenine
LC: Liquid chromatography
LC-MS/MS: Liquid chromatography – tandem mass spectrometry
MS: Mass spectrometry
NMDA: N-Methyl-D-aspartate
PHQ-9: Patient Health Questionnaire
SERT: Serotonin transporter
SSRI: Selective serotonin reuptake inhibitor
TDO: Tryptophan 2,3-dioxygenase
TNFα: Tumour necrosis factor-α
TPH: Tryptophan hydroxylase
TRP: Tryptophan
