# Supplementary material for: Tryptophan metabolism, its relation to inflammation and stress markers and association with psychological and cognitive functioning: Tasmanian Chronic Kidney Disease pilot study
Source: BMC Nephrol. 2016 Nov 10;17:171. doi: 10.1186/s12882-016-0387-3 (PMC5103367; doi:10.1186/s12882-016-0387-3)
Supplement: Additional file 2: — Chromatography and mass spectrometry conditions. (PDF 276 kb) [file 12882_2016_387_MOESM2_ESM.pdf]

<u>Supplementary Information: Chromatography and mass spectrometry conditions</u>

1.Chromatography conditions:

The autosampler was kept at 10°C, and 20µL of each prepared serum sample was loaded to the analytical column maintained at 30°C in a column oven.

Mobile phase A was 0.1% (v/v) Formic acid in water and B was 0.1% (v/v) Formic acid in Acetonitrile, and the flow rate was 0.3mL/min (typical to the "solvent saver" column in use). Table 1 describes the eluent gradient. The long run was necessary to elute and clean the column from more hydrophobic and highly-retained compounds in the serum samples, such as fatty acids, sterols etc.

Table 1. Chromatography gradient conditions:

Time (min)	Eluent A	Eluent B
0.0	95%	5%
2.5	95%	5%
5.0	70%	30%
18.0	0%	100%
23.0	0%	100%
24.0	95%	5%
30.0	95%	5%

2.Mass Spectrometry conditions:

Electrospray ionisation was used in the positive ion mode (apart from two analytes) with the following parameters: capillary voltage 2.6 kV; desolvation temperature 450°C; desolvation flow 950 L/hr; Cone flow 50 L/hr. The cone voltage and collision energy were optimised for each MRM transition as described in Table 2. MRM transitions were grouped into time windows for MS/MS analysis allowing dwell times to be maintained at >28 msec per transition.

Table 2. Multiple Reaction Monitoring (MRM):

Analyte	Retention time (min)	Ion mode	Precursor m/z	Product m/z (quantitation and validation channels)		Cone voltage (V)	Collision energy (V)	
				Product 1	Product 2		Product 1	Product 2
Neopterin	2.71	+	254	236	206	38	18	18
Quinolinic acid	3.39	-	166	122	-	23	13	-
Serotonin	6.06	+	177	160	115	15	12	25
Kynurenine	7.30	+	209	94	146	23	15	18
Tryptophan	8.09	+	205	188	146	17	10	16
Xanthurenic acid	8.10	+	206	178	132	34	18	23
3-OH Anthranilic acid	8.22	+	154	136	108	18	15	21
Kynurenic acid	8.46	+	190	162	-	32	18	-
Quinaldic acid	8.63	+	174	146	128	34	15	17
5-OH indole acetic acid	8.78	+	192	146	117	25	15	27
Indoxyl sulfate	8.82	-	212	132	80	34	18	18
Indole-3-acetic acid	10.96	+	176	130	-	26	15	-
Cortisol	11.58	+	363	121	327	32	28	18
